# Linking Environmental Genotoxins to Neurodegenerative Diseases Through Transcriptional Mutagenesis

**DOI:** 10.3390/ijms252111429

**Published:** 2024-10-24

**Authors:** Bert M. Verheijen, Marc Vermulst

**Affiliations:** 1 Department of Systems Biology, Harvard Medical School, Boston, MA 02115, USA; 2 School of Gerontology, University of Southern California, Los Angeles, CA 90089, USA

**Keywords:** DNA damage, transcription errors, mutagenesis, mutant proteins, protein aggregation, neurodegenerative diseases

## Abstract

Numerous lines of evidence suggest that DNA damage contributes to the initiation, progression, and severity of neurodegenerative diseases. However, the molecular mechanisms responsible for this relationship remain unclear. This review integrates historical data with contemporary findings to propose that DNA damage exacerbates neurodegenerative diseases by inducing transcription errors. First, we describe the scientific rationale and basic biological concepts that underpin this hypothesis. Then, we provide epidemiological, cellular, and molecular data to support this idea, and we describe new and recently published observations that suggest that the former high incidence of neurodegenerative disease in Guam may have been driven by DNA damage-induced transcription errors. Finally, we explore the long-term implications of these findings on our understanding of the impact of genotoxic stress on human aging and disease.

## 1. Introduction

Environmental toxins have been implicated in an increasing number of age-related neurodegenerative diseases that are characterized by protein misfolding and protein aggregation, including Alzheimer’s disease (AD), Parkinson’s disease (PD), and amyotrophic lateral sclerosis (ALS) [1,2,3]. For example, AD is characterized by protein deposits constructed of amyloid-ß (Aß) peptides and tau [4], while PD patients and ALS patients (in most cases) carry aggregates that are constructed of α-synuclein [5] and TDP-43 [6], respectively. The commonality of protein aggregation across multiple diseases suggests that environmental toxins may impact the aggregation process itself, rather than the biology of any disease-related protein. Understanding the molecular mechanisms that are responsible for this observation could significantly improve our ability to mitigate the impact of these diseases on society.

A common feature shared among many toxins that promote protein aggregation-associated neurodegeneration is their ability to induce DNA damage. For example, farmers that were exposed to the DNA-damaging pesticides rotenone and paraquat showed an increased risk for AD and PD compared to those that were not [7,8,9]. Similarly, cigarette smoke, car exhaust, and combustion products like benzo[a]pyrene are closely linked to AD and PD and are potent DNA-damaging agents as well [10,11,12]. DNA damage is typically associated with the induction of mutations. If these mutations were to produce proteins that exhibit amyloid- or prion-like properties, it is possible that they could explain the link between DNA damage and the development of neurodegenerative diseases. However, the fixation of DNA damage into mutations generally depends on DNA replication. Since neurons are post-mitotic cells that do not divide or replicate their DNA, they are unlikely to accumulate enough mutations to produce significant amounts of harmful proteins.

**Figure 1:**
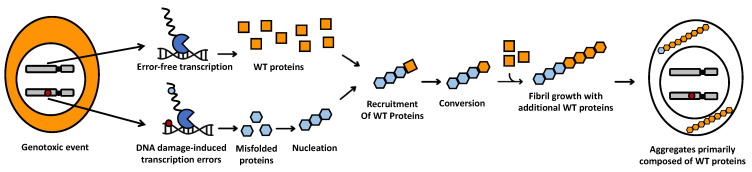
Mechanism proposed to give rise to the protein aggregates characteristic of amyloid- and prion-like diseases. After a genotoxic event, transcription errors (small blue circle) are made on damaged DNA templates (small red circle) by RNA polymerases (blue “Pacman”). These errors give rise to a limited number of mutant proteins with enhanced amyloid- or prion-like properties (blue honeycombs) that convert WT proteins (orange squares) to an amyloid- or prion-like shape. Converted WT proteins (orange honeycombs) then recruit additional WT proteins to the amyloid seed to perpetuate fibril growth without the need for further errors.

Importantly, though, DNA damage can also result in mutant proteins through transcriptional mutagenesis (TM). This process involves the production of mutant RNA molecules from wild-type (WT) DNA templates [13]. RNA polymerases rarely misincorporate a base during RNA synthesis [14,15,16], but DNA damage greatly increases the likelihood of mistakes [15,17,18,19,20]. These transcription errors are particularly important in human neurons because unlike DNA replication, transcription is an ongoing process, which means that DNA damage almost exclusively produces mutant proteins through TM. Although the number of mutant proteins that can be created by TM is limited, the impact of mutant proteins with increased amyloid- or prion-like potential can be significant. After all, amyloid- and prion-like proteins are characterized by their ability to “replicate themselves” by binding to WT proteins of the same amino acid sequence, forcing them to adopt the amyloid state [21]. These proteins can then recruit additional WT proteins to the amyloid deposit, effectively generating the large protein aggregates that characterize amyloid- and prion-like diseases (Figure 1).

Based on these considerations, we propose that transcription errors play an important role in a wide variety of neurological disorders that are caused by amyloid- and prion-like proteins. If true, this idea represents a paradigm shift in environmental health sciences because it adds a new layer of complexity to the biological effects of environmental toxins. In this review, we summarize the historical observations and contemporary research that supports this idea, describe a unique syndrome that may be associated with TM, and speculate on the long-term consequences of a role for TM in human aging and disease.

## 2. Transcriptional Mutagenesis in Neurological Disorders

The first evidence to suggest that TM plays a role in neurological disorders was provided by the late Dr. Fred W. van Leeuwen, who identified transcription errors in post-mortem brain samples of patients with non-familial cases of AD. While examining the genes that encode the ubiquitin B (UBB) and amyloid precursor protein (APP), Dr. van Leeuwen discovered that RNA polymerase II (RNAPII) tends to slip on dinucleotide repeats that are present in these genes, leading to the deletion of two bases from the transcript [22,23]. The resulting frameshifts generated truncated versions of the UBB and APP proteins (known as UBB^+1^ and APP^+1^, respectively) that were detected in the pathological hallmarks that are characteristic of the disease, suggesting that they play a role in disease progression. Further studies by Dr. van Leeuwen and others demonstrated that the UBB^+1^ protein impairs the proteasome’s ability to degrade misfolded proteins [24,25], providing a mechanistic basis for this relationship. Remarkably, it was recently discovered that expression of UBB^+1^ induced AD-like pathology in human neuronal cultures in the absence of known AD mutations [26], and that frameshift errors arise more frequently in neurons compared to other cell types [14]. Finally, the UBB^+1^ protein is also present in protein aggregates from other neurodegenerative disorders [27,28], suggesting that transcription errors could play a role in multiple neurodegenerative diseases.

**Figure 2:**
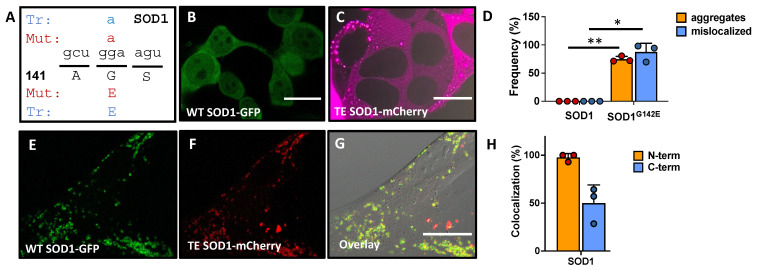
Transcription errors give rise to proteins with increased amyloid behavior. (**A**) A transcription error (Tr) was identified in the SOD1 transcript that mimics a mutation (Mut) implicated in amyotrophic lateral sclerosis. This error substituted a guanine for an adenine base, resulting in substitution of a glycine (G) for a glutamic acid residue (E) in the protein. (**B**) SOD1 is soluble and present throughout the cell, including the nucleus. (**C**) In contrast, SOD1 proteins made from erroneous transcripts form aggregates that excluded the nucleus. (**D**) Quantification of SOD1 aggregation and mislocalization. * *p* < 0.05; ** *p* < 0.01. (**E**–**G**) When WT and transcript error (TE)-derived SOD1 are expressed simultaneously; erroneous SOD1 corrupts WT SOD1 and recruits it into extranuclear aggregates. (**H**) Quantification of SOD1 colocalization with N-terminal or C-terminal tags. Scale bars 30 μm (**B**,**C**); 20 μm (**E**–**G**). Figure adapted from [29].

Importantly, TM is not limited to the UBB and APP genes. Ultra-accurate RNA sequencing of human stem cells, brain organoids, and neurons recently showed that TM is ubiquitous throughout the human transcriptome [14,15,29]. These experiments showed that transcription errors can alter the sequence of any transcript, including those encoding proteins that are implicated in neurodegenerative diseases. A subset of these errors is depicted in Table 1. One of the most intriguing observations from this table is that some transcription errors created mutant proteins that were already known to be responsible for familial cases of neurodegeneration (column 3). For example, a total of six transcription errors (column 4) were detected in transcripts that code for the SOD1 protein. One of these errors created a mutant SOD1 protein (SOD1^G142E^) that had previously been shown to cause ALS in a family that harbors a genetic mutation in the SOD1 gene. Although the number of mutant proteins created by this transcript error is limited, subsequent experiments showed that these mutant proteins were capable of converting WT proteins to an amyloid-like state, suggesting that even the limited number of mutant proteins generated by a transcription error could be sufficient to initiate long-term protein aggregation (Figure 2). Similar transcription errors were found in transcripts implicated in other neurodegenerative diseases (Table 1, [29]). For example, 57 transcription errors were detected in transcripts that code for the TTR protein, 4 of which create mutant proteins that were previously shown to cause familial cases of transthyretin amyloidosis.

However, most transcription errors arose in transcripts that are not directly involved in neurodegenerative diseases. Interestingly though, even these random errors can contribute to disease progression [30]. While studying yeast cells that display error-prone transcription, it was found that random errors often compromise the structural integrity of proteins, leading to widespread protein misfolding. Individually, these misfolded proteins may have little effect on cellular health, but collectively they can overload the protein quality control machinery, preventing the degradation of highly toxic proteins that are normally targeted by this system. For example, proteins such as TDP-43 (primarily associated with ALS), Aβ1-42 (involved in AD), Htt103Q (implicated in Huntington’s disease), and RNQ1 (a prion in budding yeast) all exhibit greater aggregation and toxicity in cells that display error prone transcription compared to WT cells [30]. Importantly, human cells that exhibit error-prone transcription also display increased protein aggregation [29]. These findings suggest that even transcription errors in unrelated transcripts can have a profound impact on neurodegenerative diseases.

In summary, these observations suggest that transcription errors can contribute to protein aggregation through three distinct mechanisms. First, they can produce highly specific mutant proteins that are directly implicated in disease [29], such as toxic Aβ and SOD1 proteins that display amyloid and prion-like properties. Second, they can impair the protein quality control machinery responsible for degrading these toxic proteins, as exemplified by the truncated UBB^+1^ protein. And third, they can overwhelm the protein quality control machinery by generating numerous randomly misfolded proteins, thereby creating the conditions that allow toxic proteins to persist and seed aggregates inside cells.

## 3. Genotoxic Stress and Transcriptional Mutagenesis

One of the most potent sources of TM is DNA damage [17]. The steric and chemical alterations caused by DNA damage can change the base pairing properties of DNA, allowing mismatched bases to form stable hydrogen bonds with each other. Because RNA polymerases rely on these base pairing properties for their fidelity, they tend to make mistakes when transcribing damaged DNA templates. For example, while RNAPII incorporates the wrong base opposite guanine once every 100,000 attempts under normal conditions, it misincorporates uracil opposite a damaged base like O^6^-methyl-guanine (O^6^-meG) once every two attempts. This stark difference highlights the significant role DNA damage plays in inducing transcription errors.

The impact of DNA damage on TM is further amplified by the bursty nature of transcription. To synthesize a large number of proteins in a short period of time, many genes are transcribed by multiple RNA polymerases at once. However, if one of these genes contains a DNA lesion, each RNA polymerase could make the same mistake at the damaged site, resulting in multiple transcripts that encode identical mutant proteins [13,18,20,29,31]. This is particularly concerning if the mutant protein has amyloid- or prion-like properties. In such cases, the simultaneous production of these mutant proteins can lead to the formation of sizable amyloid seeds, which can initiate the aggregation of misfolded proteins within the cell. Thus, the bursty nature of transcription not only accelerates protein production but also magnifies the effects of DNA damage, potentially leading to widespread cellular dysfunction.

Preventing DNA damage from inducing mutant proteins through TM hinges on effective DNA repair mechanisms. Consistent with this idea, growing evidence links DNA repair deficiencies to amyloid diseases. For example, female patients with non-familial cases of AD were recently shown to display hypermethylation of the *MGMT* promoter [32], while male patients were not. Importantly, MGMT is the DNA repair protein responsible for the repair of O^6^-meG, which is a common form of DNA damage in the brain that is also induced by environmental mutagens. Intriguingly, the MGMT promoter has one full and two half estrogen-responsive elements, which allows estrogen to upregulate MGMT expression and estrogen receptor antagonists to inhibit MGMT activity [33]. As a result, females are more likely to lose MGMT activity with age compared to males, especially at the onset of menopause when estrogen levels tend to drop. This reduced DNA repair efficiency may lead to an accumulation of O^6^-meG lesions, which would increase and prolong TM at sites of damaged bases. If so, females may produce increased numbers of amyloid- and prion-like proteins with age, potentially explaining why females are twice as likely to develop AD compared to males. Consistent with this hypothesis, we recently showed that cells that lack the ability to repair O^6^-meG display increased and prolonged TM upon mutagen exposure [29]. This connection underscores the importance of DNA repair in mitigating the impact of transcriptional errors and highlights its potential role in various protein aggregation diseases. In this context, it is important to note that sexual dimorphism in DNA damage response pathways could contribute to sex differences in the development or prognosis of other diseases as well, including cancer [34,35]. Given the impact of DNA damage on TM, it would be interesting to explore these observations in relation to TM.

Although most DNA repair pathways play a role in TM, the most important pathway might be transcription-coupled DNA repair (TCR), which is activated by a stalled or blocked RNA polymerase near a DNA lesion [36]. TCR, a specific variant of nucleotide excision repair, can remove DNA damage from the transcribed DNA strand to avoid transcriptional blockade. In doing so, it also prevents these lesions from inducing transcription errors. Some lesions are not bulky enough to pose a powerful block to RNAPII though, which limits the ability of TCR to repair them [13]. As a result, it is often the smaller DNA lesions like O^6^-meG that allow for translesion synthesis and TM. Accordingly, it is likely that environmental mutagens that create relatively small lesions that can escape detection by TCR are the most dangerous in the context of TM and neurodegeneration.

## 4. The Cycad Genotoxin Methylazoxymethanol Induces Transcriptional Mutagenesis

Perhaps one of the most compelling examples of the relationship between environmental toxins and neurodegenerative disease can be found in the Western Pacific. A neurodegenerative disorder known as amyotrophic lateral sclerosis/parkinsonism–dementia complex (ALS/PDC) was previously found to be highly prevalent in this region, particularly among the native Chamorro people of Guam (Mariana Islands) [37], Japanese residents of the Kii peninsula of Japan [38], and the Auyu and Jakai peoples of West New Guinea [39]. Although this disease is present at multiple foci in the Western Pacific, we focus here on the Guamanian cluster of ALS/PDC, because it represents the most intensively studied population of patients. Clinically speaking, ALS/PDC tends to present as a progressive motor neuron disease (ALS), parkinsonism with dementia (PDC), or a combination of both. At the neuropathological level, ALS/PDC is characterized by tau- and TDP-43-dominant multi-proteinopathy in the brain and spinal cord [40,41]. Structural analysis of tau filaments isolated from ALS/PDC brain and spinal cord tissues revealed that ALS/PDC tau primarily adopts the chronic traumatic encephalopathy (CTE) fold [42]. Importantly, no gene mutations or infectious agents have been found to be responsible for ALS/PDC in Guam [43,44], suggesting a critical role for environmental factors in disease initiation. Consistent with this hypothesis, migration studies indicate that the disease can be acquired after prolonged residence in affected geographic clusters [45] and the CTE fold of tau that is characteristic of ALS/PDC has also been detected in other disorders with an environmental etiology [46,47]. And finally, there has been a sharp decline in disease incidence coinciding with the rapid westernization of Guam [48,49], hinting at the disappearance of a critical environmental factor.

A prominent hypothesis for the cause of ALS/PDC involves exposure to toxins present in cycad plants. The Chamorro people traditionally used cycad seeds as a food source and it has been suggested that exposure to cycad and ALS/PDC in Guam are etiologically linked [50,51]. The toxicity of cycad seeds is well known, but how cycad toxicity could lead to ALS/PDC is not clear. Interestingly, one of the components of cycads, cycasin, can be metabolized to methylazoxymethanol (MAM), which is a known genotoxin. It was previously recognized that MAM can induce O^6^-meG lesions on DNA [52,53], the exact type of DNA damage found to be able to potently induce TM in experimental models [17]. It was therefore hypothesized that MAM-induced DNA lesions may promote TM in cells. We recently performed a series of experiments to test this idea [31].

**Figure 3:**
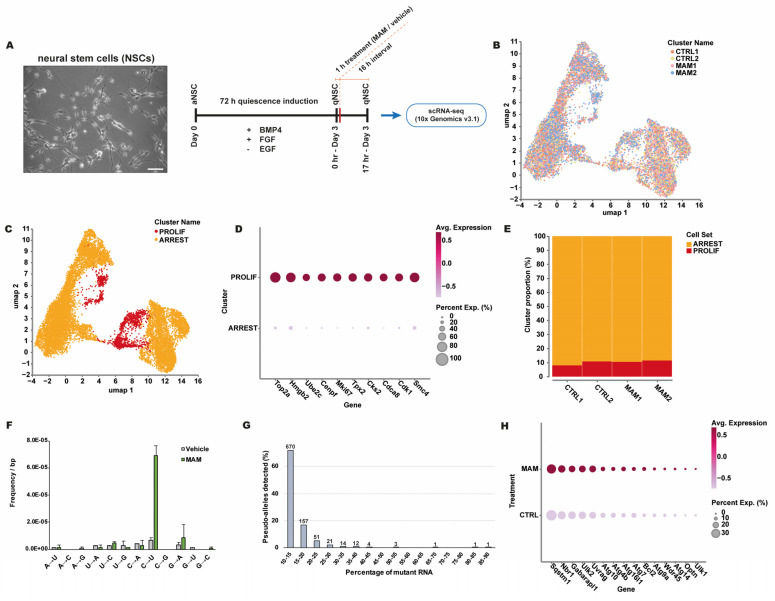
Methylazoxymethanol (MAM) induces transcriptional mutagenesis in cultured cells. (**A**) Schematic diagram of the experiment. Primary mouse hippocampal neural stem cells (NSCs) were grown in BMP4+/EGF− culturing medium for 3 days to put them in a quiescent state. Next, cells were treated with a high dose of MAM acetate (1 mM) or vehicle (phosphate-buffered saline [PBS]) for 1 h, after which they were extensively rinsed with PBS and cultured for 16 h. Cells were then collected and used immediately for single-cell RNA sequencing (scRNA-seq) experiments (10x Genomics v3.1). All cells used for experiments described in [31] were cultured at 37 °C under 5% CO_2_ and 5% O_2_. Following quiescence induction, cells were kept in BMP4+/EGF− culturing medium for treatments and 16 h intervals. The photomicrograph shows representative adherent NSCs in culture. Scale bar: 75 µm. (**B**) scRNA-seq data were processed, and individual cells were depicted in Uniform Manifold Approximation and Projection (UMAP) plots. (**C**,**D**) A transcriptionally distinct cluster of cells associated with cell proliferation genes (e.g., *Top2a* and *Mki67*) was observed. The dot plot depicts the expression of marker genes in each cluster. The diameter of the dots indicates the percentage of cells expressing the genes. This clustering was used to estimate the proportions of proliferating and arrested cells. (**E**) Bar plots depict the proportions of each cluster for each condition, which indicates that the majority of cells in the experiment were arrested. (**F**) Transcriptional error spectra in NSCs following exposure to MAM. MAM-treated NSCs showed an increased C→U error rate (average error rate: 6.9 × 10^−5^/bp) as compared to vehicle (PBS)-treated cells (average error rate: 6.7 × 10^−6^/bp). C→U errors correspond to transcriptional mutagenesis on O^6^-meG DNA lesions induced by MAM. (**G**) Analysis of pseudo-alleles (transcripts containing an error at an identical sequence location) revealed that a substantial number of repeated transcription errors occurred in MAM-treated cells (analysis of all MAM-treated cells combined). Only alleles with more than 10% mutant RNA were included. Numbers above bars indicate number of pseudo-alleles detected. (**H**) Comparison of arrested MAM-treated cells and arrested control (vehicle-treated) cells indicated that expression of transcriptional markers for autophagy induction [54] was upregulated in MAM-treated cells, although most of these changes were not statistically significant (pseudobulk limma-voom workflow). Source data were from [31] (note that in source the notation [expected sequencing read: A/C/G/T] → [interpretation of observed read: A/C/G/U] was used for transcript error spectra). Abbreviations aNSC: activated NSC; BMP4: bone morphogenetic protein 4; EGF: epidermal growth factor; FGF: fibroblast growth factor-basic; qNSC: quiescent NSC.

In these experiments, cultured mouse neural stem cells (NSCs) were chemically arrested, treated with MAM, and subsequently used for single-cell RNA sequencing (scRNA-seq) assays (Figure 3A). We reasoned that MAM-induced DNA damage could act as a template for repeated transcription errors and therefore result in the synthesis of a pool of RNAs that carry identical errors at the location corresponding to the DNA lesion site. We refer to these sites as “pseudo-alleles”, based on their ability to create both WT and mutant RNA molecules. In a scRNA-seq experiment, it should be possible to detect such events, because transcripts in a single cell correspond to the same (damaged) genome. Using non-replicating cells (Figure 3B–E) was important to prevent the fixation of DNA damage into mutations, which would confound transcript error measurements. We found that the C→U error rate was significantly higher in MAM-treated cells compared to control cells (Figure 3F). C→U lesions are the primary transcription error induced by O^6^-meG lesions and thus consistent with MAM-induced DNA damage [17]. Importantly, we identified a considerable number of pseudo-alleles in MAM-treated cells (Figure 3G), strongly suggesting that MAM induces TM. We then confirmed this observation with CirSeq, a powerful, ultra-accurate RNA sequencing approach [31].

Based on the concepts described above, it seems plausible that MAM-induced TM could hold significance for ALS/PDC pathogenesis. MAM may promote the TM-mediated generation of amyloid- and prion-like proteins that drive the disease [55]. Additionally, increased transcription error output could provide conditions that potentiate toxic proteins and disease progression. Gene expression comparisons between MAM-treated cells and control cells provided some clues for autophagy induction by MAM (Figure 3H), an indicator of proteotoxic stress, although this remains to be validated in follow-up experiments. Saliently, transcript error-derived proteins (i.e., UBB^+1^) and accumulated protein quality control factors were previously found in ALS/PDC post-mortem brain tissues [56,57,58,59]. Although a causal role for cycad in the etiology of ALS/PDC remains unproven [60,61,62], ALS/PDC may represent a prototypical environmental neurodegenerative disease linked to transcription errors.

## 5. Discussion

The ideas presented here could have profound implications for our understanding of the relationship between genetic toxicology and neurodegenerative diseases. First, they propose a novel mechanism by which genotoxic agents promote the development of diseases characterized by protein aggregation. By inducing transcription errors, genotoxic agents can directly influence the production of disease-related amyloid- and prion-like proteins. These mutant proteins can then convert WT proteins to an amyloid state as well and seed the aggregates that are characteristic of neurodegenerative diseases. If so, the production of mutant proteins could provide a unified pathophysiological mechanism for both inherited and non-inherited protein aggregation diseases; whereas inherited cases are caused by mutant proteins that result from genetic mutations, non-inherited cases are caused by mutant proteins that result from non-genetic mutations (i.e., transcription errors). This process is further facilitated by transcription errors that affect unrelated proteins, which can impair and overwhelm the protein quality control machinery that is responsible for counteracting these aggregates [30]. This dual impact underscores the potential impact of TM on neurodegenerative diseases. In doing so, TM could complement or amplify other mechanisms proposed to contribute to the production of amyloid proteins, including “spontaneous” protein misfolding and aggregation, mistakes made during protein translation and folding [63], or direct damage to macromolecules (e.g., protein oxidation).

Second, most genotoxic agents have been identified by their ability to induce genetic mutations. It is likely, though, that many of these mutagenic agents also induce transcription errors. Consequently, we may have underestimated the impact of mutagenic agents on human health. Concentrations of mutagens that were previously deemed to be safe may pose a risk to human health after all, due to their ability to induce transcription errors. Thus, it would be prudent to re-evaluate mutagens for their impact on transcriptional fidelity, potentially with a modified version of the Ames test that incorporates transcript error measurements. Assaying compounds that are known to induce small DNA lesions that can escape TCR may be prioritized, especially if they create alkylating damage like O^6^-meG [64]. Some of these compounds include environmental mutagens like NDMA and commonly used chemotherapeutics like temozolomide. Other high-priority toxins include those that were previously linked to neurodegenerative diseases, namely, paraquat and rotenone, two pesticides that create various forms of oxidative DNA damage, including 8-oxo-guanine and uracil lesions, which are both potent sources of TM [13]. For similar reasons, benzo[a]pyrenes, which are released during combustion reactions, may also represent high-priority candidates for screens. It is possible, though, that these screens will not identify every possible candidate. It has been observed that some mutagenic agents, like methyl methanesulfonate (MMS), do not induce transcription errors. It is conceivable, therefore, that an opposite chemical may also exist—a genotoxic agent that induces transcription errors without causing genetic mutations. Such chemicals might be present in our environment, consumables, and drugs without our knowledge. The improved Ames test proposed here could be instrumental in identifying these previously undetected chemicals, thereby enhancing our understanding of their potential health risks.

Finally, the effects of transcription errors induced by genotoxic agents may extend well beyond the boundaries of neurodegenerative diseases. Protein aggregation does not only play a role in neurodegenerative diseases, but many other age-related diseases as well [65,66,67,68], including myopathy, heart disease, kidney disease, and cancer [69]. For instance, >15% of human cancers carry amyloid deposits of TP53 [70], c-Abl [71], the Von Hippel–Lindau protein [72], or MEF-2 [73]. Thus, DNA damage-induced TM could potentially impact a wide array of diseases. In some cases, the impact of genotoxic agents on transcriptional fidelity may also impact the basic biology of human cells, without altering a specific disease process. For example, transcription errors could alter cell fate [74], or the function of extremely long-lived proteins [75] and RNA molecules [76] that persist inside cells for years. Furthermore, it is possible that the unique proteins generated by these errors might be perceived as foreign by the immune system, triggering unnecessary and potentially harmful immune responses.

To test these hypotheses, the development of new technology and experimental animal models is urgently needed. In particular, the creation of pre-clinical mouse models that exhibit error-prone transcription could provide invaluable insights into the impact of transcription errors on mammalian health. For example, one strategy would be to knock out fidelity factors that control the error rate of transcription in vivo. These models are expected to show increased susceptibility to neurodegenerative diseases characterized by protein aggregation, which would help us understand the direct consequences of TM in vivo. Additionally, pre-existing mouse models or human cell lines that display DNA repair deficiencies could be re-examined from the perspective of TM to yield critical information. For example, mice that are deficient for MGMT or TCR could be interesting targets for such an analysis [77,78]. Such models might reveal previously unrecognized connections between DNA damage, transcription errors, and disease progression. Together, these innovative models and approaches could usher in a new era of genotoxic research, significantly advancing our understanding of neurodegenerative diseases and potentially uncovering novel therapeutic targets.

## 6. Conclusions

Recent studies demonstrate that TM can generate aberrant proteins that display enhanced amyloid- and prion-like properties. As such, TM may contribute to various protein aggregation disorders, including age-related neurodegenerative diseases. Historically, a lack of adequate detection tools has precluded comprehensive and systematic analyses of the role that TM plays in these disorders. However, the recent development of new and improved transcript error detection tools has made it possible to explore the impact of TM on neurodegenerative diseases in unprecedented detail. As a result, we strongly encourage researchers in the fields of genetic toxicology and neurodegeneration to assess the ability of toxins to induce TM, and then to correlate these measurements with the impact of these toxins on protein aggregation and human cognition. Together, these experiments could allow detailed studies, meta-analyses, and large cohort studies to tease apart the link between DNA damage and neurodegenerative diseases, so that we can better understand their origins and take rational precautions to curb their impact on society.

## Figures and Tables

**Table 1:** Transcription errors affect proteins directly implicated in amyloid and prion diseases. Column 1: Gene name. Column 2: Protein symbol. Column 3: Disease associated with protein. Column 4: Number of errors detected in transcripts that were derived from this gene. Column 5: Number of errors that generate mutant proteins identical to those seen in familial cases of amyloid diseases. Column 6: Number of errors that affect an amino acid (aa) known to be involved in disease but mutate it to a different residue compared to the clinic. ALS: amyotrophic lateral sclerosis. CJDL Creutzfeldt–Jakob disease. CMT: Charcot–Marie–Tooth disease. EPM1: progressive myoclonic epilepsy type 1. FAF: familial amyloidosis, Finnish type. FBD: familial British dementia. FDD: familial Danish dementia. FFI: fatal familial insomnia. GSS: Gerstmann–Straussler–Scheinker disease. Table modified from [29]. The source paper provides additional details.

Gene	Protein	Disease	Errors Detected	Mutations Mimicked	Key aa Affected
ABri peptide	ITM2B	FBD and FDD	66	2	6
Amyloid Precursor Protein	APP	Alzheimer’s disease	266	6	9
Cystatin-B	CSTB	EPM1	33	2	1
Fused in Sarcoma	FUS	ALS	25	1	3
Gamma-crystallin D	CRYGD	Coralliform cateracts	1	1	0
Gelsolin	GSL	FAF	35	1	1
Heterogeneous nuclear ribonucleoprotein D-like	HNRNPDL	Limb-girdle muscular dystrophy 1G	15	1	1
Medin	MFGE8	Cerebrovascular dysfunction	115	1	1
Neurofilament Heavy Polypeptide	NEFH	CMT and ALS	1	1	0
Prion protein	PRNP	CJD, GSS, FFI	10	1	1
Receptor-interacting serine/threonine-Protein Kinase 1	RIPK1	Neuroinflammation	1	1	0
Solute Carrier Family 3 Member 2	SLC3A2	Lysinuric protein intolerance	57	1	0
Superoxide Dismutase 1	SOD1	ALS	6	1	0
Transforming Growth Factor Beta-Induced	TGFBI	Corneal Dystrophy	701	6	12
Tumor Protein P53	TP53	Cancer	60	5	1
Transthyretin	TTR	Transthyretin Amyloidosis	57	4	9
Tubulin Alpha-1A Chain	TUBA1A	Tubulinopathies	149	3	15

## Supplementary Materials

The following supporting information can be downloaded at: https://www.mdpi.com/article/10.3390/ijms252111429/s1. Supplementary File S1.
